# *Escherichia coli* O157:H7 *tir* 255 T > A allele strains differ in chromosomal and plasmid composition

**DOI:** 10.3389/fmicb.2023.1303387

**Published:** 2023-12-15

**Authors:** Margaret D. Weinroth, Michael L. Clawson, Gregory P. Harhay, Mark Eppinger, Dayna M. Harhay, Timothy P. L. Smith, James L. Bono

**Affiliations:** ^1^Department of Molecular Microbiology and Immunology, USDA ARS Meat Animal Research Center, Clay Center, NE, United States; ^2^South Texas Center for Emerging Infectious Diseases, San Antonio, TX, United States

**Keywords:** whole genome sequencing (WGS), comparative genomics, bacterial genomics, STEC, Plasmids, Bacteriophage, Translocated Intimin Receptor

## Abstract

Shiga toxin-producing *Escherichia coli* (STEC) O157:H7 strains with the T allele in the translocated intimin receptor polymorphism (*tir*) 255 A > T gene associate with human disease more than strains with an A allele; however, the allele is not thought to be the direct cause of this difference. We sequenced a diverse set of STEC O157:H7 strains (26% A allele, 74% T allele) to identify linked differences that might underlie disease association. The average chromosome and pO157 plasmid size and gene content were significantly greater within the *tir* 255 A allele strains. Eighteen coding sequences were unique to *tir* 255 A allele chromosomes, and three were unique to *tir* 255 T allele chromosomes. There also were non-pO157 plasmids that were unique to each *tir* 255 allele variant. The overall average number of prophages did not differ between *tir* 255 allele strains; however, there were different types between the strains. Genomic and mobile element variation linked to the *tir* 255 polymorphism may account for the increased frequency of the T allele isolates in human disease.

## Introduction

Shiga toxin-producing *Escherichia coli* (STEC) O157:H7 is a foodborne pathogen that causes illnesses ranging from mild self-limiting cases to hemolytic uremic syndrome (Slutsker et al., 1997). A 2011 assessment estimated that it causes 63,153 illnesses, 2,138 hospitalizations, and 20 deaths annually in the United States (Scallan et al., 2011). Cattle are recognized as a major reservoir for this pathogen (Smith et al., 2001), although it has been isolated from other animals such as wildlife (deer and birds), other domestic livestock (sheep and goats), and companion animals (dogs), as well as from non-animal sources such as vegetables and environmental samples (Mead and Griffin, 1998).

STEC O157:H7 is a relatively recently emerged pathogen with the first reported case linked to it in the 1980s (Mead and Griffin, 1998; Karch et al., 1999). Evolutionary models have described the emergence of this serotype via a stepwise evolution from O55:H7 chromosomes (Wick et al., 2005). STEC O157:H7 strains harbor a highly conserved non-conjugative F-like plasmid known as pO157, ranging between 92 and 104 kilobases (kb) in length (Lim et al., 2010) that has been linked to chromosome evolution (Nyong et al., 2020). While several proteins encoded on it have been associated with virulence, there is still uncertainty around the role pO157 plays in infection and overall virulence (Lim et al., 2010).

STEC O157:H7 genomes may carry many different mobile genetic elements (MGEs) in addition to pO157. Other plasmids include pEC4115, which is a 37 kb conjugal transfer plasmid (Eppinger et al., 2011), pColD157, which is a 6,675 bp colicin-containing plasmid (Hofinger et al., 1998), and pOSAK1, which is a 3,306 kb plasmid-containing ColE1-like replication system (Makino et al., 1998). Another major type of MGE found in the STEC O157:H7 genome is integrated bacteriophages (prophages). The genomes of STEC O157:H7 contain an average of 11.9% prophages that are classified as lambda-like, P4-like, and unclassified (Bobay et al., 2013). Some prophages contain Shiga toxin genes which encode for a significant virulence factor of STEC O157:H7 and have integrated into several known integration sites in the genome (Recktenwald and Schmidt, 2002; Bielaszewska et al., 2006; Serra-Moreno et al., 2007; González-Escalona et al., 2019). Insertion sequence (IS) element IS*629* is the most abundant IS element in STEC O157:H7 and has been shown to be responsible for genome diversification (Ooka et al., 2009a; Rump et al., 2011b). IS*629* has also been used to develop typing methods for STEC O157:H7 (Ooka et al., 2009b; Yokoyama et al., 2011; Stanton et al., 2014; Kameyama et al., 2015; Toro et al., 2015). MGEs are also a source of antibiotic resistance genes (ARGs) in STEC O157:H7. The prevalence of ARGs in STEC O157:H7 is generally low and ranges from 10% to 14.7% (Schroeder et al., 2002; Greig et al., 2023). Interestingly, ARGs can be located on both chromosomes and plasmids in STEC O157:H7 (Greig et al., 2023).

Several studies have found that not all STEC O157:H7 subtypes are equally likely to cause disease in humans (Kim et al., 1999; Bono et al., 2007; Manning et al., 2008; Whitworth et al., 2010). Octamer-based genome scanning of STEC O157 strains showed a biased distribution of isolates based on clade distributions with most human isolates being found in clade I compared to clade II that had more non-human isolates (Kim et al., 1999). Genotyping of enterohemorrhagic *E. coli* strains identified 96 SNPs that cluster isolates into nine clades (Manning et al., 2008). The Manning clade eight strains were responsible for more severe disease in humans and were seven times more likely to cause hemolytic uremic syndrome.

The ability of STEC O157:H7 to cause disease is driven by its attachment to intestinal epithelium via the products of a pathogenicity island in the genome known as the locus of enterocyte effacement (LEE) and the production of Shiga toxins (Kaper et al., 2004). The translocated intimin receptor gene *tir*, found in the LEE, has a single-nucleotide polymorphism (SNP; *tir* 255 T > A), with strains having the T allele being 34 times more likely to cause disease in humans (Bono et al., 2007). However, strains isolated from diseased humans with the *tir* 255 A allele suggest that the non-synonymous SNP, while valuable as a marker for disease association, is not itself responsible for the lower propensity to cause disease. In fact, the SNP does not appear to affect *Tir* protein activity, and *tir* 255 A allele strains are able to cause attaching and effacing lesions (Besser et al., 2007). Thus, genetic differences responsible for the association of the *tir* 255 T allele with human disease have not as yet been identified.

Whole-genome sequencing (WGS) of bacterial genomes has allowed a high level of discernment between strains in research and public health settings (Didelot et al., 2012; Rusconi et al., 2016; Nadon et al., 2017; Tagini and Greub, 2017; Jenkins et al., 2019). Bacterial genomes can be comprehensively analyzed for SNPs within genes of interest and macro-differences with WGS. Short-read sequencing technologies have limited ability to resolve complex genomes with large inversions and repeated phage regions such as STEC O157:H7 (Koren et al., 2013; Koren and Phillippy, 2015; De Maio et al., 2019). In contrast, long-read sequencing technologies can bridge repetitive regions, providing a better and more accurate understanding of genome structure. Integrating long-read sequencing as scaffolding with short reads to polish the assembly and find smaller plasmids can produce a higher quality genome (De Maio et al., 2019). This level of assembly gives the best chance of finding genetic determinants that may be responsible for *tir* 255 T allele strains associating with human disease more than A allele strains. The objectives of this study were to use the hybrid short- and long-read sequencing strategy on a collection of STEC O157:H7 strains to characterize the genetic diversity and compare the genomes of strains with either the T or A allele at *tir* 255. These comparisons provide a more complete understanding of this important public health pathogen and new insight into the genomic features that contribute to the enhanced association of *tir* 255 T allele strains with human disease.

## Materials and methods

### Bacterial strain selection

A total of 58 *E. coli* O157:H7 strains [isolated from cattle (*n* = 28), deer (*n* = 1)], ground beef (*n* = 6), and humans (*n* = 23) were included in this study for phylogenetic and comparative genome analyses (Supplementary Table 1). The strains were mainly isolated in the United States (*n* = 50), but some were included from Japan (*n* = 3), Scotland (*n* = 2), Germany (*n* = 1), and Denmark (*n* = 1), with one of unknown origin. Forty-eight strains were sequenced based on their tir 255 genotype and their diverse distribution across a previously described phylogenetic classification method based on polymorphism-derived genotypes as described by Bono et al. (2012). Within the 48 sequenced strains, approximately the same relative proportion of *tir* 255 A genotype (13/48 = 31%) and *tir* 255 T genotype (33/48 = 61%) strains were used as described from the polymorphism-derived genotyping system [*tir* 255 A strains (56/175 = 32%) and genotype *tir* 255 T strains (119/175 = 68%); Bono et al., 2012]. Ten complete closed genomes with the *tir* 255 T genotypes were downloaded from the National Center for Biotechnology Information (NCBI) database and were used for all analyses except those on the distribution of transposable insertion sequence.

### Genome sequencing, genotyping, and annotation

Strains frozen in glycerol were struck onto CHROMagar O157 plates (DRG International, Mountainside, NJ) and incubated overnight at 37°C. The following day strains were inoculated into 1 mL of LB and grown overnight at 37°C with shaking. The next morning, 250 ul of the overnight culture was added to 10 mL of LB and incubated at 37°C with shaking for 3.5 h before undergoing DNA extraction. DNA was extracted as described (Clawson et al., 2009) using Qiagen Genomic-tip 100/G columns (Valencia, CA, United States) and quantified using a nanodrop and a Quantus Fluorometer with QuantiFluor dye (Promega, Madison, WI, United States). An aliquot of DNA (10 μg) was sheared to a 30 kb target fragment length using g-TUBEs (Covaris, Woburn, MA, United States) and concentrated with 0.45x volume AMPure PB beads (Pacific Biosciences, Menlo Park, CA, United States), as per manufacturer’s instruction. Sheared and concentrated DNA (5 μg) was used to make sequencing libraries using the SMRTbell Template Prep Kit 1.0 according to the manufacturer’s protocol. Fragments ≥ 15 kb were selected using the BluePippin (Sage Science, Beverly, MA, United States). The library was bound with polymerase P6 for sequencing on a Pacific BioSciences (Pacific Biosciences) RS II sequencing platform with chemistry C4 and the 360-min data collection protocol.

Another aliquot of the same DNA preparation used for long-read sequencing was used for short-read library construction. One microgram of DNA was sheared to 350 bp fragment size using a microTUBE AFA Fiber Pre-Slit Snap-Cap 6x16mm (Corvaris, Woburn, MA). Libraries were constructed using the TruSeq DNA PCR-Free HT Library Preparation kit (Illumina, Inc., San Diego, CA) and quantitated using the KAPA Library Quantification Kit (F. Hoffmann-La Roche Ltd., Basel, Switzerland). Libraries were pooled into groups of 24 and run on an Illumina MiSeq with the MiSeq Reagent Kit v3 (600 cycles).

Raw PacBio reads were assembled using HGAP (Chin et al., 2013) in SMRT analysis v8.0 and contigs imported into Geneious v2019.1(Biomatters Ltd., Auckland, New Zealand). If present, overlapping sequence on the ends of the contigs was removed from the 5′ and 3′ ends to generate circularized chromosomes and plasmids. Closed chromosomes were reoriented after finding a putative origin of replication with Ori-Finder 2 (Luo et al., 2014) for the chromosome of one strain and then orienting all subsequent chromosomes to that same starting position. The closed chromosomes and plasmids were polished twice for accuracy using the RS_Resequencing v1.0 protocol in SMRT analysis by mapping PacBio reads to the chromosomes and plasmids. Further error correction was performed using the short-read data via Pilon v1.23 (Walker et al., 2014). Finally, short reads were mapped to the chromosome and assembled plasmids using Geneious Mapper (Biomatters, Ltd.), and unused reads were *de novo* assembled using the Geneious assembler (Biomatters, Ltd.) for the identification of additional plasmids. All genomes and plasmids were annotated with the NCBI Prokaryotic Genome Annotation Pipeline v4.8 (Tatusova et al., 2016). The genomes were previously genotyped for the *tir* 255 allele (Bono et al., 2007), a 175 polymorphism-derived genotype (Bono et al., 2012), and the Shiga toxin-encoding bacteriophage insertion site (Besser et al., 2007; Whitworth et al., 2008). Clade typing was performed according to Manning et al. (2008). Clades and subgroups were assigned by *in silico* interrogation of the allelic status of 89 core genome SNPs in the assembled genomes using a custom workflow on Galaxy (Giardine et al., 2005; Goecks et al., 2010), which was informed by eight definitive polymorphic positions (Riordan et al., 2008; Yokoyama et al., 2012). VirulenceFinder v2.0.3 (Camacho et al., 2009; Joensen et al., 2014; Malberg Tetzschner et al., 2020) was used to type the Shiga toxin variants.

### Phylogenetic tree construction and linear fragment visualization.

A phylogenetic tree was constructed from all O157 chromosomes with Parsnp v1.6.2 (Treangen et al., 2014) with the “-c” flag to include all files with strain 493/89 as the root because it is proposed to be the oldest *E. coli* O157:H7 ancestor. FigTree v1.4.4 (Rambaut, 2009) was used for tree visualization, and Evolview v3 (Subramanian et al., 2019) was used to annotate tree branches and leaves, as well as the origin of the isolates. EasyFig v2.2.2 (Sullivan et al., 2011) was used to visualize linear comparisons of multiple genomic loci insertions, while individual gene annotations were visualized in SnapGene Viewer v5.2 (GSL Biotech, LLC.).

### Core and pangenome

The fasta files from all isolates were exported from Geneious (Biomatters, Ltd.) and annotated with Prokka v1.14.5 (Seemann, 2014) using the—proteins option with the annotated GenBank file from STEC O157:H7 isolate TX 376-2 (GenBank CP038287–CP038289) for annotation. The .gff files from the Prokka annotation were used with Roary v3.13.0 (Page et al., 2015) to define the core and pangenome of the 58 isolates. The gene_presence_absence.csv from the Roary analysis and the *tir* 255 A vs. T trait .csv file were used as input into Scoary v1.6.16 to look for associations of components of the pangenome with the *tir* 255 alleles. The .grr file from the Parsnp analysis of the STEC O157 genomes containing all variant sites was converted to a .vcf file using Harvest tools (Treangen et al., 2014). The .vcf file was imported into PLINK v1.90 (Chang et al., 2015) and the linkage disequilibrium based variant pruner option “--indep-pairwise” with a window size the entire length of all SNPs identified with an r^2^ = 0.99 (and visually inspected to assure r^2^ = 1) was used to reduce all SNPs to just those that were informative. Linkage disequilibrium SNP associations were visualized with the LinkageMapView v2.1.2 (Ouellette et al., 2018) in R v3.6.

### Identification of prophage insertion sites

Prophages were identified using the PHASTER API (Arndt et al., 2016). The number of phages present (including complete, intact, and questionable) and percentage of the chromosome associated with prophages (computed by dividing the total region length of all prophages associated with a chromosome by the total chromosome size) were compared between strains, source of isolate, and *tir* 255 allele using the SAS Studio v3.8 PROC MIXED procedure with the LSMeans statement with pdiff and cl options at an alpha = 0.05.

The stx-containing prophages were extracted with the flanking core chromosomal genes using Geneious (Biomatters, Ltd.). The extracted prophages were grouped according to their insertion sites and aligned using the Mauve (Darling et al., 2004) plugin in Geneious (Biomatters, Ltd.). Shiga toxin genes were extracted from the prophage sequences, grouped according to subtype, and aligned using the Geneious alignment tool in Geneious (Biomatters, Ltd.).

### Evaluation of insertion sequence elements

The insertion sequence (IS) elements from the chromosome and their corresponding pO157 plasmids were extracted from their GenBank annotations using Geneious. The genomes from the 10 closed genomes downloaded from GenBank were not used in this study because they were annotated with a different version of the NCBI prokaryotic genome annotation pipeline. The IS elements were grouped into families, and the number of transposases per family was used in the analysis.

### Antibiotic resistance gene identification

Antibiotic resistance genes were identified using the Comprehensive Antibiotic Resistance Database (CARD; Alcock et al., 2020) resistance gene identifier using perfect (100% sequence similarity) and strict (≥80% sequence similarity) hits only and excluding nudges.

### pO157 plasmid analysis

The relatedness of 56 pO157 plasmids was determined using Parsnp v1.2 (Treangen et al., 2014) with the Sakai pO157 plasmid as the reference and visualized with Gingr v1.3 (Treangen et al., 2014). pO157 plasmids from strains LSU61 and 493/89 were excluded from this analysis because their pO157 plasmids are approximately 30 kb larger and are missing the known virulence gene *toxB* (Nyong et al., 2020). Comparison of the phylogenetic trees of the pO157 plasmids to their respective chromosomes was done with the .tree files from Parsnp using the tanglegram option from Dendroscope v3.7.1 (Scornavacca et al., 2011; Huson and Scornavacca, 2012). Plasmids were grouped based on similarities using the Mauve (Darling et al., 2004) plugin in Geneious (Biomatters, Ltd.).

### Description of other plasmids identified in the genome

Non-pO157 plasmids were grouped according to size in Geneious (Biomatters, Ltd.). Visual inspection of the gene annotations from each size group was used to further refine the grouping into those with similar gene annotations. The plasmids grouped by size and annotation were compared using the Mauve (Darling et al., 2004) plugin in Geneious (Biomatters, Ltd.).

## Results and discussion

### Sequencing and assembly

The chromosome from MB9-1 was the only example of the 48 strains sequenced that could not be closed due to the presence of two ~60 kb duplicated regions that could not be resolved with the long reads produced by the RSII. Thirty-one plasmids smaller than 30 kb were identified by assembling Illumina reads that failed to map to the long-read-derived genomes. Information about each genome is available in Supplementary Table 1. The isolates had an average chromosome length of 5,507,515 bp with a range from 5,362,528 bp to 5,643,409 bp and an average of 1.7 plasmids (ranging from 1 to 4, with size ranging from 1.5 to 145.3 kb). The average number of protein-coding sequences (CDS) within strains was 5,633 and ranged from 5,393 to 5,868.

### Genome comparisons of *tir* 255 allele strains

Genomes were compared between strains carrying either the *tir* 255 T or *tir* 255 A allele. Strains with the *tir* A allele chromosomes were found to have larger chromosomes (*p =* 0.015) than *tir* T chromosomes (means 5,539,556 vs. 5,496,221 bp, respectively [95% C.I. 5,509,981 bp to 5,569,131 bp and 5,478,332 bp to 5,514,109 bp]); the pO157 plasmid size was also larger on average (*p <* 0.001, *tir* A mean = 95,890 bp, [95% C.I. 95,019 bp to 96,761 bp] *tir* T mean = 93,013 bp [95% C.I. 92,486 bp to 93,539 bp]). Similarly, strains with the *tir* A allele genomes had a greater number of CDS when compared to strains with the *tir* T genomes (means 5,701 vs. 5,601, respectively).

**Figure 1:**
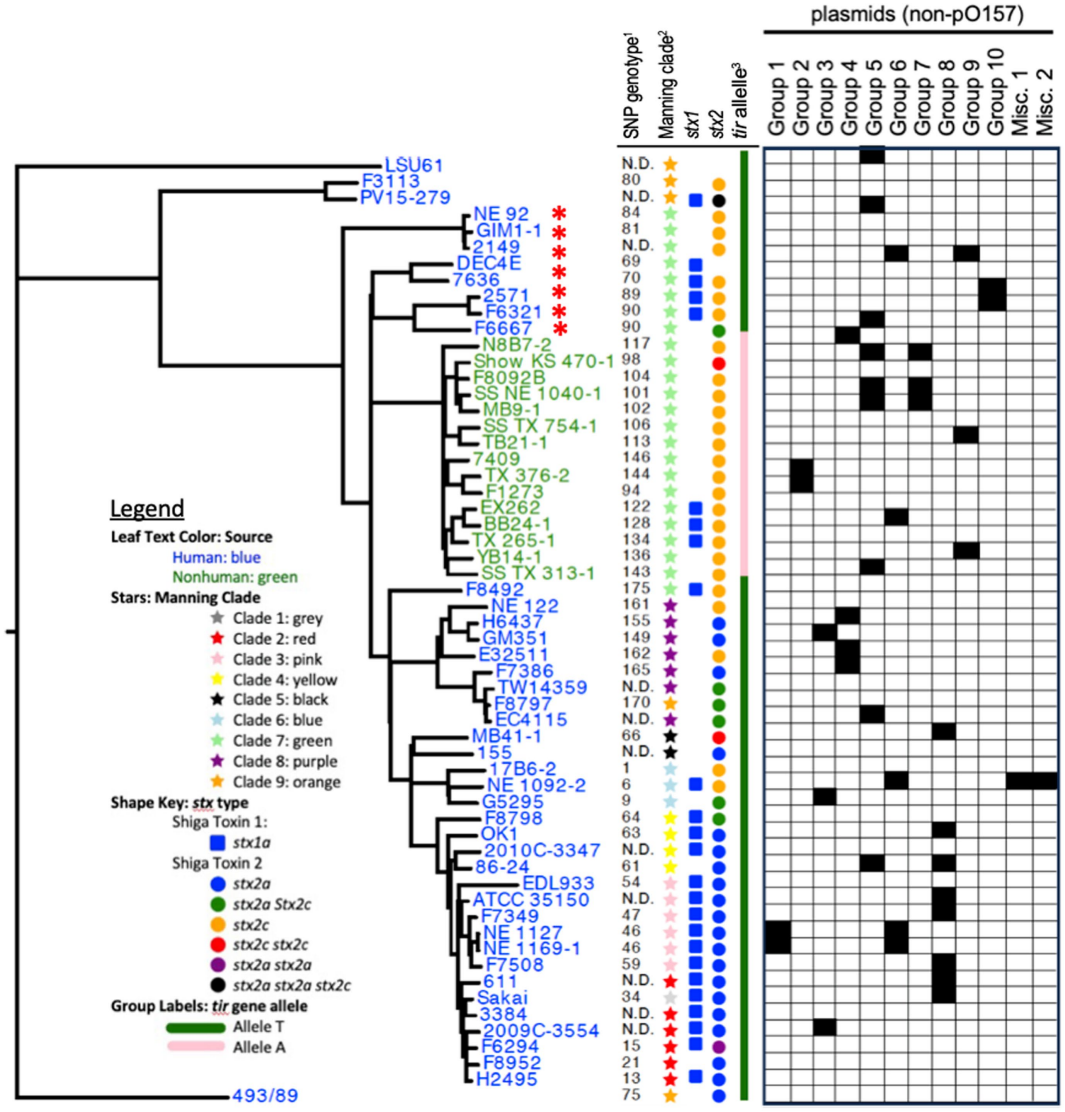
Phylogenetic tree of all STEC O157:H7 chromosomes included in the study. Leaf text colors indicate the source of the isolate as human (blue) or non-human (green). Leaf decorations and labels are based on previously published SNP-based phylogeny (^1^Manning et al., 2008, ^2^Bono et al., 2012), *tir* 255 allele genotype (^3^Bono et al., 2007), and Shiga toxin subtype and variant as determined by VirulenceFinder (Camacho et al., 2009; Joensen et al., 2014; Malberg Tetzschner et al., 2020). N.D. abbreviation is for not determined. The non-pO157 plasmids are separated into plasmid groups across the top of the columns. The black boxes beneath the group name indicate that the plasmid was found in the corresponding strain. The red asterisk indicates strains that were not found to contain the mdfA gene by CARD because of a missense mutation resulting in a truncated and presumably inactive protein.

These results are consistent with previous studies that show an association of a smaller genome size in pathogenic bacteria than their nearest neighbors that are non- or less-pathogenic (Ochman and Moran, 2001; Moran, 2002; Toft and Andersson, 2010; Weinert and Welch, 2017). This reduction in genome size of pathogenic strains of bacteria is mainly seen between bacteria species and is rarely seen within a single bacterial species (Weinert and Welch, 2017). However, *Streptococcus suis*, an emerging pathogen in swine, shows a reduction in genome size that is associated with its ability to cause disease more often than expected by chance (Murray et al., 2021). The loss of mobile genetic elements was responsible for a large portion of the reduced genomes with the remaining reductions located in the other genomic elements (Murray et al., 2021). Here, the increased size of the *tir* A allele genomes and increased number of genes could be from the gain of mobile genetic elements, but prophages do not appear to be responsible for this as there is not a significant difference in the number of integrated prophages between *tir* A and *tir* T allele genomes. The most parsimonious explanation for the reduced association of *tir* A allele strains with human disease is that their predecessor acquired genetic material that reduced fitness or propensity to cause disease in humans. Alternatively, a less parsimonious explanation is that the *tir* T allele strains may have reduced genome size in multiple events, and not as a part of continuous evolution because the *tir* A allele strains are flanked by *tir* T allele genomes on both the ancestral and progeny branches of the phylogenetic tree (Figure 1).

### Phylogenetic tree clusters with Shiga toxin content from *tir* 255 T allele and *tir* 255 A allele strains

The phylogenetic tree of core genomes from the 58 strains resembled the trees from which the strains were chosen according to their polymorphism-derived genotypes (Bono et al., 2012) and the Manning clades (Manning et al., 2008; Figure 1). As seen previously, the *tir* 255 A and *tir* 255 T allele strains group separately on the tree as previously described, with only one of the *tir* 255 A allele strains isolated from a human source while the rest were from a non-human source. The human and non-human sources of *tir* 255 T allele strains were disbursed throughout the tree with no association with clusters.

Shiga toxin genes were also found both on their own and in combination. However, LSU 61, a proposed evolutionary intermediate strain, was the only strain to not contain a Shiga toxin gene in its genome (Nyong et al., 2020). In addition, DEC 4E was the only strain with no *stx2* gene in its genome. The *stx1_a_* gene was found in 25 of the strains, while 28 had the *stx2_a_* gene and 35 had the *stx2_c_* gene. Thirty-five strains contained multiple *stx* genes with the most common combination being *stx1_a_* and *stx2_a_* (*n* = 13), followed by *stx1_a_* and *stx2_c_* (*n* = 8), *stx2_a_* and *stx2_c_* (*n* = 5), *stx2_c_* and *stx2_c_* (*n* = 2), *stx1_a_*, *stx2_a_*, and *stx2_a_* (*n* = 1), *stx1_a_*, *stx2_a_*, and *stx2_c_* (*n* = 1), and lastly *stx1_a_*, *stx2_a_*, *stx2_a_*, and *stx2_c_* (*n* = 1; Figure 1). The *stx2_c_* gene in STEC O157:H7 strain MB41-1 had an IS*629* element inserted into the Shiga toxin subunit b gene presumably causing the Shiga toxin protein to be inactivated. Disruption of *stx2* genes by IS element has been previously reported. The *stx2_c_* subunit A (Jinneman et al., 2000; Loftsdóttir et al., 2017; Nyong et al., 2020) and subunit B (Kusumoto et al., 1999) genes and the *stx2_a_* subunit A gene (Fitzgerald et al., 2019) were disrupted by different IS elements including IS*1203*, IS*629*, and IS*66*. Disruption of the *stx2* gene has been identified in different countries and different regions of the genes indicating the potential for IS elements to integrate into any strain, and there does not appear to be a targeted sequence for IS element integration into the *stx* genes (Kusumoto et al., 1999; Loftsdóttir et al., 2017; Fitzgerald et al., 2019). Strains with a *stx* gene disrupted by an IS element are a potential risk to human health as IS elements can excise from the gene leaving behind an intact *stx* gene capable of making toxins (Kusumoto et al., 2001; Loftsdóttir et al., 2017). The *tir* 255 A allele strains only contained the *stx2_c_* gene variant, while the *tir* 255 T allele contained both the *stx2_a_* and *stx2_c_* variants. Both *tir* 255 A and *tir* 255 T allele strains contained *stx1_a_* although *tir* 255 T allele strains had a higher proportion (22/43 = 51%) than the *tir* 255 A allele strains (3/15 = 20%; Figure 1; Supplementary Table 1).

### Some CDS and SNP-linked alleles associated with only one *tir* 255 SNP allele

The core STEC O157:H7 chromosome was composed of 4,091 coding sequences (CDS) while the pangenome encompassed 10,079 CDS, with 1,789 CDS being singletons. Chromosomes had on average 5,392 identifiable CDS (range 5,153 to 5,612), or 76% (4,091/5,392) of the chromosome being conserved among the STEC O157:H7 analyzed in this study. Of the singletons identified, the chromosomes contained an average of 30 (range 2 to 232) unique genes. Three strains LSU61, 493/89, and PV15-279 accounted for 25.6% (*n* = 458/1,789) of the singletons (Supplementary Table 2).

There were 18 CDS unique to the *tir* 255 A allele chromosomes and three CDS unique to the *tir* 255 T allele chromosomes (Supplementary Table 3). Specific genes in *tir* 255 T allele chromosomes encode sel1 repeat family protein (*n* = 1), RHS repeat protein (*n* = 1), and phage tail protein (*n* = 1). Sel1 repeat proteins have been associated with virulence in *Legionella pneumophila* though when investigated in the more closely related *Klebsiella pneumoniae*, their function was not characterized (Lery et al., 2014). The *tir* 255 A allele-specific genes encode *AlpA* family transcriptional regulator (*n* = 1), Clp protease *ClpB* (*n* = 1), DNA cytosine methyltransferase (*n* = 1), glycerophosphodiester phosphodiesterase (*n* = 1), host cell division inhibitor Icd-like protein (*n* = 1), hypothetical proteins (*n* = 6), IS*3* family transposase (n = 2), IS*66* family insertion sequence hypothetical protein (*n* = 1), phage polarity suppression protein (*n* = 1), phage receptor (*n* = 1), phage tail protein (*n* = 1), and a DNA primase (*n* = 1).

Analysis for SNPs in linkage disequilibrium with the *tir* 255 alleles that could contribute to the ability of a strain to cause human disease revealed 204 SNPs on the chromosome and two on the pO157 plasmid. In total, 101 were non-synonymous and 62 were synonymous, with seven resulting in amber mutations and 32 residing in intergenic regions (Supplementary Table 4). The amber mutations were in the glycerophosphodiester phosphodiesterase gene (*glpQ*), ethanolamine ammonia-lyase reactivase gene (*eutA*), aquaporin water channel gene (*apqZ*), a fimbrial protein precursor CDS (*lpfE*), two hypothetical genes and a bacteriophage N4 receptor, and outer membrane subunit gene. While it is difficult to speculate on the function of the truncation of the hypothetical genes and bacteriophage to reduce the ability of *tir* 255 A alleles to cause disease in humans, the remaining four truncated genes have metabolic significance. *glpQ* is periplasmic and can catalyze the hydrolysis of glycerophosphodiesters. This enzyme allows the cell to use a variety of glycerophosphodiesters in the *glp* system (Larson et al., 1983). *eutA* is the sole enzyme responsible for reactivating ethanolamine ammonia-lyase (EAL). EAL is induced in the presence of both ethanolamine and vitamin B_12_ and is involved with both anaerobic fermentation and catabolism (Mori et al., 2004). *apqZ* prompts changes to the environmental osmolality by allowing the flux of water and is required for rapidly growing cells (Calamita, 2000). *lpfE* is a predicted fimbriae subunit protein and is found in the *lpf1* operon in STEC O157:H7. Its proposed ligands are fibronectin, collagen IV, and laminin (McWilliams and Torres, 2014).

One of the 32 linked SNPs in the intergenic regions was found in the disrupted coding sequence for the propionyl-CoA:succinate CoA transferase (*scpC*) gene that was truncated previously by a 28 or 56 bp indel. The *scpC* indel was found in 13 of the 15 *tir* 255 A allele strains but was not found in any *tir* 255 T allele strains. Upon further investigation, the *scpB* gene just upstream of *scpC* also contained an indel that is 10 bp in length. All 15 *tir* 255 A allele strains have this indel, while it was not found in any *tir* 255 T allele strains. *scpB* and *scpC* are two of three genes in the succinate to propionate conversion pathway, one of three known pathways for propionate utilization and the only one found in STEC O157:H7. Propionate is an abundant volatile fatty acid that is produced by gut microbiota and found in high concentrations in the mammalian gastrointestinal tract. It has an antimicrobial activity (Salmond et al., 1984) and can block a number of metabolic pathways in *Salmonella enterica* (Horswill et al., 2001). To combat the negative effects of propionate, several pathways are used by bacteria to catabolize this compound into a carbon and energy source. The indels in the *scbB* and *scbC* genes appear to inactivate the propionate to succinate conversion and remove the beneficial properties of this pathway to STEC O157:H7 in the mammalian gut.

### Prophage content of *tir* 255 allele chromosomes

There were 1,144 prophage regions identified in the 58 chromosomes analyzed that could be categorized into 33 different types (Table 1). Chromosomes had an average of 20 prophages (range = 16 to 36). There was no statistical difference (*p* = 0.07) between *tir* 255 allele strains when comparing the number of prophages. Seven of the 33 different phage sequences were found in similar percentages between the *tir* 255 A or T allele strains, while 16 were found more abundant in the *tir* 255 T allele strains, and 10 were commonly observed in the *tir* 255 A allele strains (Supplementary Table 5). There were nine prophages that were identified in all the *tir* 255 A allele strains, while only one was observed in all the *tir* 255 T allele strains, although this number is likely somewhat impacted by differing strain number representation. Three other prophages were only found in the *tir* 255 A allele strains, while 11 were found only in the *tir* 255 T allele strains. Twelve of the fourteen *tir* 255 strains contained less than five allele-specific prophages with most of them clustering with related strains. One *tir* 255 T allele-specific prophage, PHAGE_Entero_SfI_NC_027339, was found in 67% (29/43) strains. PHAGE_Entero_SfV_NC_003444 was the most specific prophage for *tir* 255 A allele strains with 87% of the strains containing this prophage compared to only 7% of the *tir* 255 T allele strains. *Enterobacteria* phage BP-4795 was the only prophage that was common to all strains; it has been characterized to contain two IS*629* elements genes associated with type III secretion (Creuzburg et al., 2005). Additionally, two prophages (Stx2-converting phage 1717 and *Shigella* phage POCJ13) were found in 96.6% (56/58) of the strains. The human isolates did show statistical differences in phage content, and the small magnitude of these differences may not be of biological interest, a conclusion that is further supported by the fact that there is not a specific phage associated with only human-associated strains.

**Table 1:** Prophages identified within all *Escherichia coli* O157:H7 genomes and the levels of their completeness.

Most similar phage	Accession number	Incomplete^a^	Questionable	Intact	Total
Enterobacteria phage BP-4795^*^	NC_004813	1 (10)	19 (8 to 15)	277 (11 to 55)	297 (8 to 55)
Stx2-converting phage 1717	NC_011357	6 (3 to 5)	41 (3 to 10)	67 (3 to 63)	114 (3 to 63)
Enterobacteria phage YYZ-2008	NC_011356	7 (2 to 4)	38 (4 to 9)	61 (15 to 52)	106 (2 to 52)
Enterobacteria phage cdtI	NC_009514	-	-	65 (11 to 26)	65 (11 to 26)
Enterobacteria phage DE3	NC_042057	12 (2 to 2)	34 (11 to 25)	12 (11 to 22)	58 (2 to 25)
Enterobacteria phage P88	NC_026014	-	-	57 (14 to 36)	57 (14 to 36)
Shigella phage POCJ13	NC_025434	57 (5 to 6)	-	-	57 (5 to 6)
Acinetobacter phage vB AbaM ME3	NC_041884	11 (3 to 3)	40 (3 to 4)	-	51 (3 to 4)
Enterobacteria phage Sf6	NC_005344	48 (4)	-	-	48 (4)
Enterobacteria phage mEp460	NC_019716	35 (9)	2 (9 to 10)	5 (13 to 24)	42 (9 to 24)
Enterobacteria phage lambda	NC_001416	2 (19 to 24)	21 (24 to 26)	9 (17 to 25)	32 (17 to 26)
Enterobacteria phage SfI	NC_027339	-	27 (5)	3 (5)	30 (5)
Enterobacteria phage P4	NC_001609	8 (2 to 6)	3 (2 to 6)	18 (9 to 10)	29 (2 to 10)
Phage Gifsy-1	NC_010392	-	24 (3)	-	24 (3)
Salmonella phage 118970 sal3	NC_031940	-	8 (4 to 5)	16 (3 to 4)	24 (3 to 5)
Escherichia phage pro483	NC_028943	1 (4)	1 (3)	20 (27 to 30)	22 (3 to 30)
Enterobacteria phage Mu	NC_000929	-	-	19 (33 to 34)	19 (33 to 34)
Enterobacteria phage SfV	NC_003444	-	-	16 (34 to 37)	16 (34 to 37)
Enterobacteria phage 933 W	NC_000924	-	1 (45)	10 (50 to 69)	11 (45 to 69)
Stx2 converting phage II DNA	NC_004914	2 (2)	1 (48)	6 (65 to 66)	9 (2 to 66)
Synechococcus phage S-SKS1	NC_020851	8 (2)	-	-	8 (2)
Enterobacteria phage VT2phi 272	NC_028656	-	-	5 (39 to 60)	5 (39 to 60)
Escherichia phage P13374	NC_018846	4 (2)	-	-	4 (2 to 2)
Escherichia phage PA28	NC_041935	-	-	4 (53 to 55)	4 (53 to 55)
Bacillus phage Shanette	NC_028983	2 (2)	-	-	2 (2 to 2)
Shigella phage SfII	NC_021857	-	-	2 (20 to 21)	2 (20 to 21)
Stx2 converting phage vB EcoP 24B	NC_027984	-	-	2 (30)	2 (30)
Enterobacteria phage fiAA91-ss	NC_022750	-	-	1 (29)	1 (29)
Enterobacteria phage P2	NC_001895	-	-	1 (27)	1 (27)
Enterobacteria phage SfMu	NC_027382	-	-	1 (34)	1 (34)
Escherichia phage D108	NC_013594	-	-	1 (34)	1 (34)
Salmonella phage ST64B	NC_004313	-	-	1 (3)	1 (3)
Salmonella phage vB SosS Oslo	NC_018279	-	-	1 (19)	1 (19)
Total		204 (2 to 24)	260 (2 to 48)	680 (3 to 69)	1,144 (2 to 69)

^*^Asterisks indicate a phage was found in all genomes. ^a^The number of strains followed in parenthesis by the highest number of proteins most similar to those in the region.

An average of 817 kb of each chromosome was comprised of prophage regions, which corresponds to an average of 14.8% (range 11.4% to 27.7%) of the chromosome. There was no difference when prophage percentages were compared between *tir* 255 alleles (*p* = 0.248). Bobay et al. previously observed the chromosome of STEC O157:H7 strain EC4115 to be composed of 13.5% prophage with the average chromosomal content of prophage in the five compared STEC O157:H7 strains to be 11.9% (Bobay et al., 2013). A similar study in *E. coli* O26:H11 found that the prophage content of those genomes ranged between 8.7% and 16.7%. While a review of the genes within these prophage regions was mostly non-specific (with the majority of annotations attributed to hypothetical genes), past study has suggested that these regions contribute to survivability and virulence (Delannoy et al., 2017).

Prophages that harbored *stx* genes in STEC O157:H7 strains were further scrutinized. Of the 33 prophages identified in the STEC O157:H7 strains, 10 were found to harbor at least one *stx* gene in one genome, with 66% residing in one of the three most common: Stx2-converting phage 1717 (34%), *Enterobacteria* phage BP-4795 (19%), or *Enterobacteria* phage 933 W (12%). Further characterization of these three prophages found that the phages differed substantially. *Enterobacteria* 933 W was only present while carrying *stx* genes, and only one copy of the phage per genome was present. On the other hand, both Stx2-converting 1717 and *Enterobacteria* BP-4795 were found in multiple copies within genomes (an average of 1.6 intact copies [range 1 to 2] and an average of 4.8 intact copies [range 2 to 6], respectively) and both with and without *stx* genes. From a composition standpoint, *Enterobacteria* phage BP-4795 was diverse within the population and even within a genome. Finally, Stx2-converting phage 1717 was observed in two forms (at least in some genomes), one in the form of a smaller ~20 kb prophage that does not harbor *stx* genes and the other contained in a larger ~60 kb prophage that does harbor *stx*.

### Shiga toxin-containing prophage insertion sites are more conserved in *tir* 255 A allele strains

Prophages that contained *stx* genes were found to integrate into five chromosomally encoded genes (Table 2) that were previously identified as sites for *stx*-containing prophages. All 15 *tir* 255 A allele strains had *stx2_c_* containing prophage integrated at *sbcB* with two strains harboring two *stx2_c_* containing prophage integrated into tandem at the *sbcB* integration site (Supplementary Table 6). Three of the 15 *tir* 255 A allele strains had a *stx1_a_*, *stx2_a_*, and *stx2_c_* prophage integrated at the *yehV* integration site. The *stx1_a_* containing prophage identified in *tir* 255 T allele strains was integrated into three sites on the chromosome, *yehV*, *argW*, and *sbcB*, while *stx2_a_* containing prophage was integrated into four sites on the chromosome, *argW*, *sbcB*, *wbdR*, and *yecE*. *stx2_c_* containing prophage was integrated into *sbcB* and *yehV*. Past study has shown that the *stx1* containing prophage integrates into *yehV* while *stx*2 containing prophage integrates into *wrbA* or *sbcB* (Bielaszewska et al., 2006) and *argW* is associated with *stx*1 integration (González-Escalona et al., 2019). The *yecE* gene has been described as an integration site in *E. coli* for prophage containing *Stx2e* in a ONT:H^−^ (Recktenwald and Schmidt, 2002) and *stx2* from both an *E. coli* O157:H7 (De Greve et al., 2002) and an *E. coli* O157:NM (Bielaszewska et al., 2006).

**Table 2:** Insertion sites identified for prophages containing Shiga toxin genes.

*stx* Gene	*yecE*	*argW*	*yehV*	*sbcB*	*wrbA*	Total
*stx1_a_*	--	3	21	1	--	24
*stx2_a_*	4	6	--	1	16	27
*stx2_c_*	--	1	2	35	--	37
Total	4	11	23	37	18	

While most Stx prophages were found integrated into previously described genes, this was not always the case. Only prophages integrated at *YecE* were found to harbor a single type of *stx* gene; the rest were found to have one to three types of *stx-*containing prophage integrated into specific sites (Supplementary Table 6). This is in agreement with Serra-Moreno et al. (2007) who found that while these phages have a preferred primary insertion site, a secondary site is used if the primary site is occupied. With this in mind, strains described here that fall outside of insertion site norms may provide useful insight into variation in strain evolution.

**Figure 2:**
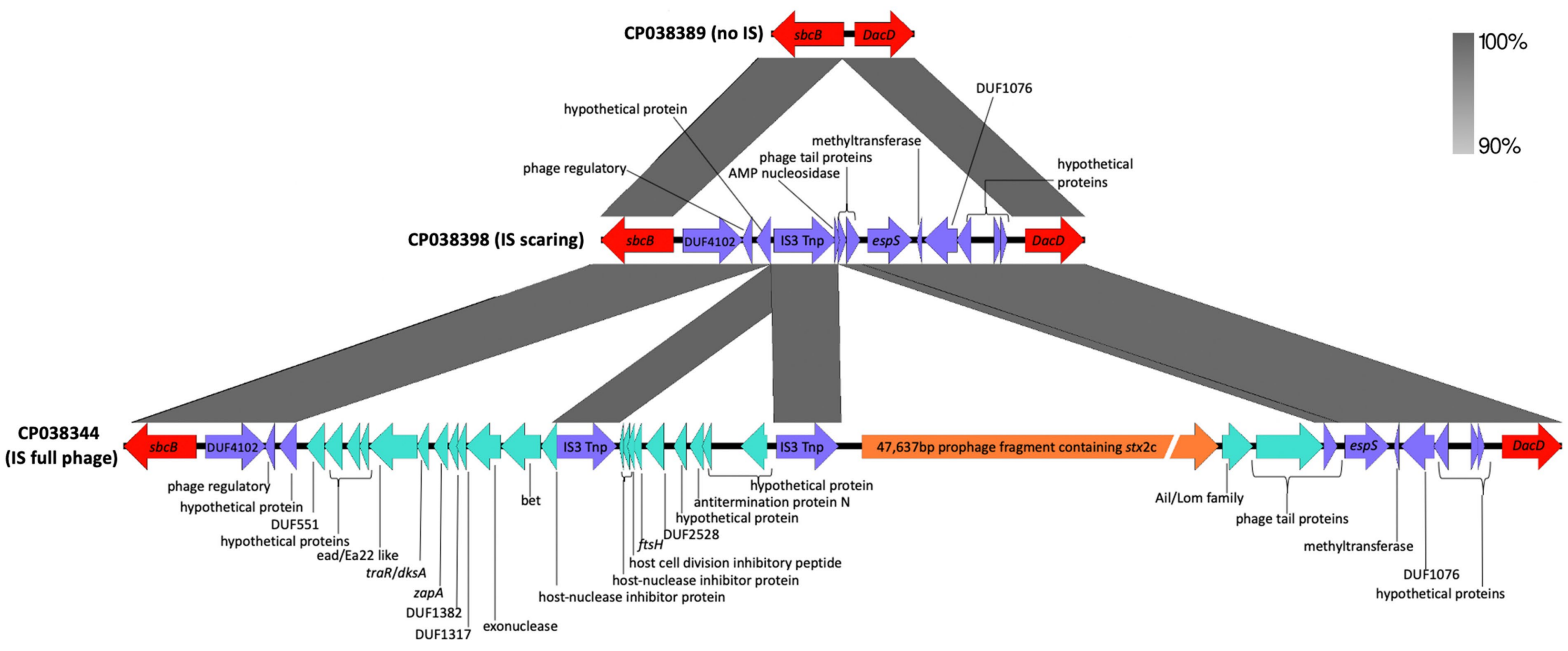
Genetic map showing the apparent scarring left by excision of a phage. The region from an ancestral *Escherichia coli* O55:H7 chromosome (CP038389) (top) did not have an insertion of a *stx2_c_* containing prophage at the *sbcB* gene. The intact *stx2_c_* contains prophage from strain Gim1-1 (CP038344) (bottom), and the genes left behind from the excision of the prophage containing the *stx2_c_* gene from strain DEC4E (CP038398) (middle) has some scarring associated with a prophage insertion and subsequent excision. The genes are represented by arrows with the red arrow representing the flanking chromosome genes and the purple arrow representing the genes shared by all three chromosomes. The green and orange arrows represent prophage genes. The shaded areas between the genetic map represent the percent similarly of the regions to each other.

Prophages can excise from genomes and integrate. When a prophage is completely excised from a genome, there typically are no remnant genes left behind. However, prophage excision can leave scars (Feiner et al., 2015), which allows for a more complete understanding of the strain’s evolutionary history. Here, prophage scarring was observed at the *sbcB* insertion site (Figure 2) when compared to an empty interion site from an O55 isolate. The scaring provides evidence of a prophage containing *stx2c* excising from the genome. This is interesting as a previous study (Asadulghani et al., 2009) has noted that most O157 phages contain one or more genetic defects and have low potential activity as mobile genetic elements (with their role being more evolutionary in STEC emergence).

### Number of insertion sequence CDS was not found to differ between *tir* 255 allele strains

The insertion sequence (IS) elements from the chromosome of 48 strains and their corresponding pO157 plasmids were extracted from their GenBank annotations using Geneious. The IS elements were grouped into families, and the number of transposases per family was used in the analysis. There was no difference in the number of IS elements in the chromosome between the *tir* 255 allele strains. The *tir* 255 T allele strains contained an average of 99 IS elements (range 54 to 123), while the *tir* 255 A allele strains contained an average of 101 (range 84 to 118). On the other hand, the *tir* 255 A allele pO157 plasmids contained an average of 18 IS elements per plasmid (range 14 to 24), while the *tir* 255 T allele strains contained an average of 14 IS elements per plasmid (range 10 to 19).

The total number of IS elements identified across the 48 chromosomes and pO157 plasmids was 5,531 with 4,793 identified in the chromosomes and 738 in pO157 plasmids (Supplementary Table 7). This averages to about 115 transposases per genome with 100 per chromosome and 15 per pO157 plasmid. The most common IS element identified on both the chromosome and pO157 was the IS*3* family transposase CDS (*n* = 1,222). The second most common, IS*66* family insertion sequence hypothetical protein CDS had only two fewer IS elements (*n* = 1,220), with the third most common IS element being the IS*66*-like element IS*Ec8* family transposase CDS (*n* = 494). While 46 of the 48 strains had the same top three chromosomal IS elements, IS*30*-like element IS*30* family transposase CDS was the most common IS element found in two ancestral strains 493–89 and LSU61. The top two most common IS elements identified on pO157 were the same as the chromosome, IS*3* family transposase CDS (*n* = 295) and IS*66* family insertion sequence hypothetical protein CDS (*n* = 104), respectively. The third most common IS element on pO157 was IS*1* family transposase CDS (*n* = 95). Overall, IS*3* family transposase CDS (*n* = 1,516) and IS*66* family insertion sequence hypothetical protein CDS (*n* = 1,324) were the most abundant IS elements in the 48 strains in this study representing 62% of all IS elements identified.

IS elements have been previously studied in STEC O157:H7, but the main focus has been on IS*629*, the most abundant IS element in STEC O157 strains. IS*629* is a non-replicative 1.31 kb region made up of *orfA* and *orfB* and belongs to the IS*3* family, and subgroup IS*51*. IS*629* has played a role in the insertional inactivation of genes including the *stx* gene (Kusumoto et al., 2004; Loftsdóttir et al., 2017; Nyong et al., 2020). We also observed the integration of an IS*629* transposase into the *stx2* beta subunit gene in strain MB41-1. The inactivation of *stx* genes by IS*629* has no apparent effect in cattle but could excise itself from the *stx* gene causing human disease with a negative PCR result (Loftsdóttir et al., 2017). Several groups have used IS*629* distribution as a way to type strains for epidemiological investigation, given the biased distribution associated with major phenotypic lineages (Ooka et al., 2009a; Yokoyama et al., 2011; Rump et al., 2011a; Stanton et al., 2014; Toro et al., 2015). The association was further enhanced by the inclusion of other STEC O157:H7 genes including *stx* and *norV* in genotyping assays (Stanton et al., 2014; Kameyama et al., 2015).

### Antimicrobial resistance genes on the chromosome were not found to differ between *tir* 255 allele strains

**Figure 3:**
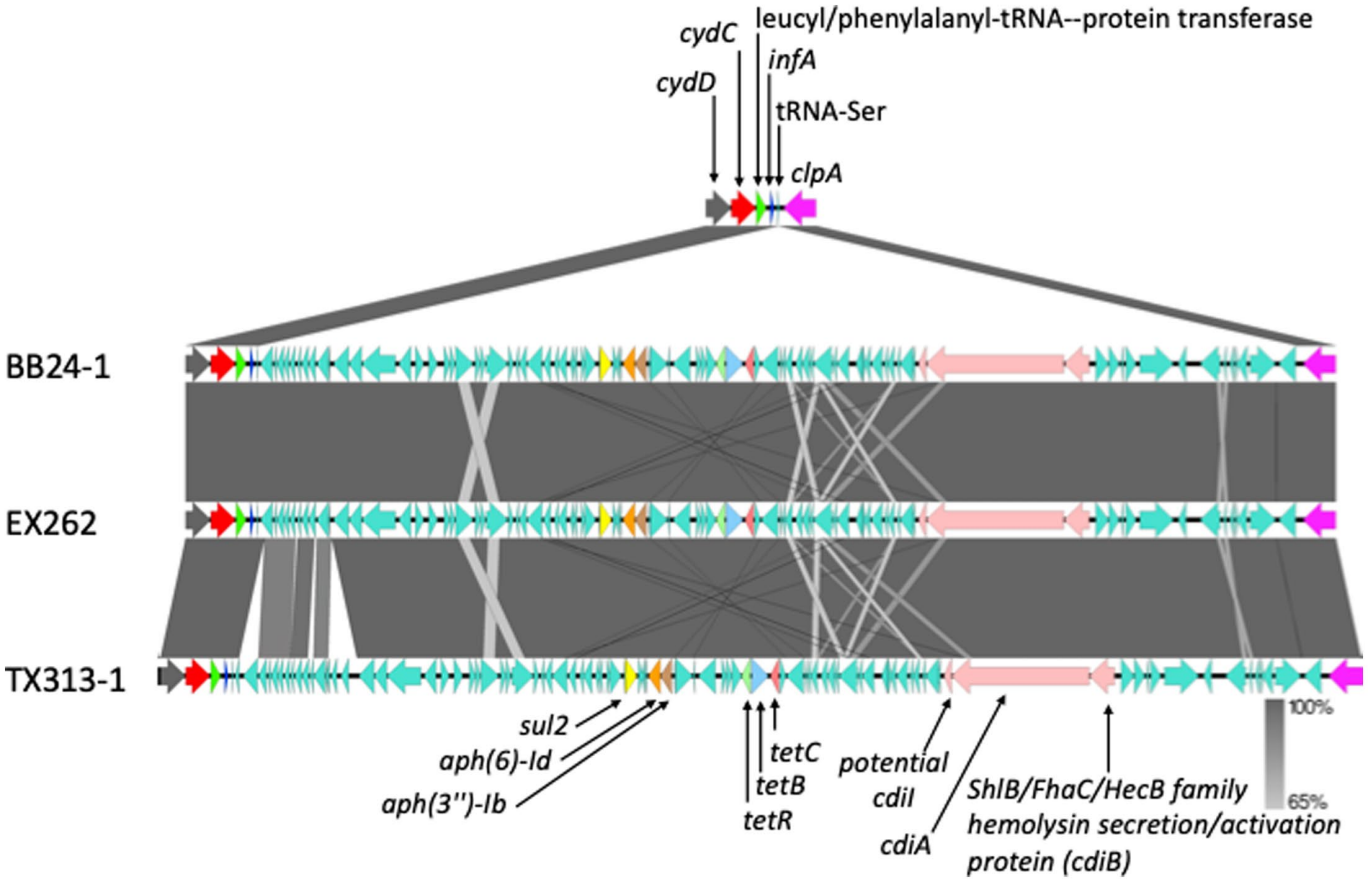
Genetic map showing an 80 kb integrated region in the chromosome from three STEC O157:H7 strains contains antibiotic resistance genes. Each colored arrow represents a different gene, except the green color which represents different genes in the inserted region. Gene names are located either above or below their respective gene. The shaded areas between the genetic map represent the percent similarly of the insertion regions to each other.

The average number of antimicrobial resistance genes (ARGs) was not different between *tir* 255 A allele strains (avg = 53.9, range 53 to 58) and *tir* 255 T allele strains (avg = 52.8, range 51 to 55). An average of 53 (range 51 to 58) resistance genes were identified within all chromosomes, and the majority of these (44 genes with elfamycin-resistant EF-Tu identified in all genomes) were conserved across all chromosomes Supplementary Table 8. This result is not surprising because many species of bacteria, including *E. coli*, are known to be intrinsically resistant to certain antibiotics (Duo et al., 2008; Cox and Wright, 2013). Efflux pumps were the most common mechanisms of resistance followed by target alteration. Nine of the 16 ARGs that were not found in all chromosomes were present in more than 86% (≥ 50/58) of them. One of the nine genes was the putative tetracycline antibiotic efflux pump, multidrug transporter *mdfA*. This gene was found in 50 of the 58 strains. Eight strains (2149, 2571, 7636, DEC4E, F6321, F6667, Gim1-1, and NE92) were not found to contain the *mdfA* gene by CARD because of a missense mutation resulting in a truncated and presumably inactive protein. These eight strains were also found grouped next to each other on the phylogenetic tree (Figure 1). A set of five ARGs occurred in three chromosomes (two aminoglycoside resistance genes [*aph(6)-Id* and *aph(3″)-Ib*], two major facilitator superfamily efflux pumps that confer resistance to tetracycline (*tetB* and *tetC*), and a target replacement gene associated with sulfonamide resistance (*sul2*; Figure 3)). The resistance genes were divided into two cassettes, one consisting of *aph(6)-Id*, *aph(3″)-Ib*, and *sul2* and the other consisting of the *tetB*, *tetC*, and *tetR*. The cassettes were part of an 80–84 kb genomic island inserted between *cycD* and *clpA*. Of the 91 genes in this genomic island, there were 22 hypothetical proteins and 11 insertion sequence/transposase genes. The three genomes with the integrated antibiotic cassettes were highly related (all *tir* 255 *A*, Manning clade 7, and containing *stx2c*). This cassette has been previously reported in STEC O157:H7 *tir* 255 A allele strains FREK944 (CP016625.1) and strain 446,541 (Greig et al., 2023), a STEC O145:H28 strain (FWSEC0002; Tyson et al., 2019), and an *E. coli* O22:H8 strain (154; Da Silva et al., 2022) that can interfere with STEC O157:H7 *in vivo* and *in vitro colonization*. The cassettes in strains FRIK944, FWSEC0002, and 446,541 are inserted between *cycD* and *clpA* and are more similar to each other than the STEC cassettes reported here. There were 14 other strains from England in addition to strain 446,541 isolated over a 4-year period that had the same ARG content. However, placement on their chromosome could not be determined because the genomes were sequenced using short-read sequencing technology. These cassettes appeared in both *tir* 255 allele strains, indicating no preference for one allele over the other, and on multiple continents, indicating that these cassettes are widely disseminated. The cassette from strain 154 is integrated between the *yagQ* and *proA* genes and has sequence similarity with ~60 kb of the other cassettes. STEC O157:H7 strain 1,090–2, *tir* 255 T allele strain, has two aminoglycoside resistance genes [*aph(6)-Id* and *aph(3″)-Ib*] flanked by a transposase and IS elements on one end and a transporter gene on the other end. This cassette was integrated into the chromosome between the *lsrK* autoinducer-2 kinase gene and an autotransporter barrel domain-containing lipoprotein gene. Tadesse et al. reported that an almost identical cassette minus the transporter gene was found on a 54 kb (CP018646 and CP018649) and 118 kb (CP018643) plasmid from *Salmonella enterica* serovar Enteritidis (Tadesse et al., 2018). These strains are historical *S. enteritidis* isolates isolated from humans in 1974 (CP018643), 1979 (CP018646), and 1981 (CP018649). STEC O157:H7 strain 1,090–2 was isolated from a steer at harvest in Nebraska in September 2000. This cassette appears to be ubiquitous and promiscuous, in nature as it is found across multiple decades and genera.

Most ARGs identified were present in all or most genomes, indicating that the resistome of *E. coli* O157:H7 seems to be stable and not to vary widely among this population. Some of the ARGs observed in the present study were associated with an insertion region, but there is little evidence to suggest that there is a diverse movement of ARGs within the STEC population. Nonetheless, this finding places no measure of likelihood on the possibility of a strain’s acquisition of ARGs within niche environments.

### Plasmid pO157 has a strong evolutionary link to the host chromosome and the *tir* 255 allele

**Figure 4:**
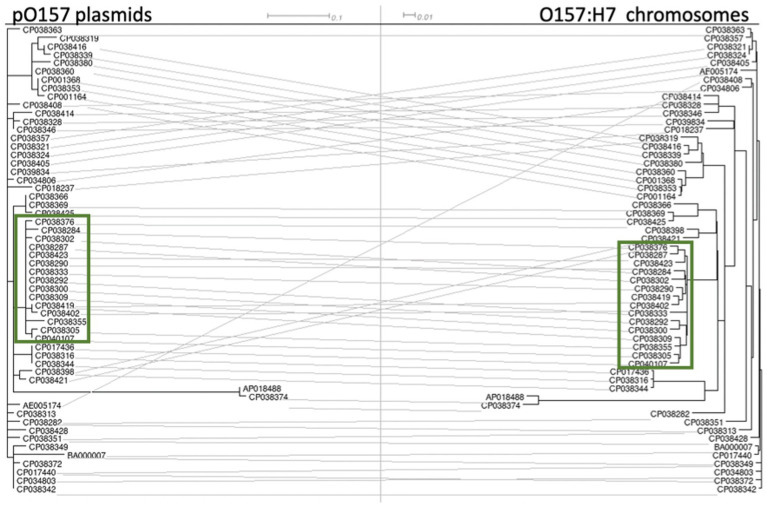
Tanglegram of pO157 plasmids (numbered with their chromosome’s GenBank accession number for ease of reading) and their STEC O157:H7 chromosome. The accession numbers boxed in green are *tir* 255 A allele strains. The accession numbers from the remaining non-highlighted strains are *tir* 255 T allele strains. The intermediate ancestral strains 493/89 and LSU 61 were not used in this analysis due to their pO157 plasmid being approximately 20 kb larger than the pO157 plasmids in other STEC O157:H7 strains.

A tanglegram constructed between 56 STEC O157:H7 chromosomes and their associated pO157 plasmids showed a clear association of groupings between the two, regardless of their *tir* 255 allele variant (Figure 4). Chromosomes and O157 plasmids fell into the same phylogenetic tree structure in nearly every case, with very few incongruences between the two trees. This is in agreement with Nyong et al. (2020) who found a stable evolutionary relationship between the host chromosome and pO157 plasmids. pO157 plasmids from strains PV15-279 and F3113 were distinct from the rest of the pO157 tree (Figure 4), and upon further inspection, this was due to a 171 bp region containing 20 SNPs. The SNPs lie outside of any coding regions and were flanked by a replication initiation protein upstream and a transposase downstream. These two strains were from disparate geographically and sample source, with one coming from a Japanese human sample (PV15-279) and a United States FSIS sample (F3113). Further inspection of this region did not identify annotations that might have provided a biological explanation for variation from the other plasmids.

There were 42 informative SNPs on the pO157 plasmid (pruned from 107 SNPs total). Two of the 42 SNPs were in linkage disequilibrium with the *tir* 255 allele. Both SNPs resulted in non-synonymous amino acid changes. The first was identified within the *tagA* gene 1,438 G > A (GenBank reference: AB011549, gene BAA31757.3) with the G allele occurring in *tir* 255 T allele strains and the A occurring with the *tir* 255 A allele strains. The second SNP was found in the *toxB* gene 7453 A > G (GenBank reference: AB011549, gene BAA31815.1) with the G allele occurring with the *tir* 255 T allele strains and the A occurring with the *tir* 255 A allele strains (Supplementary Table 4).

These two SNPs are noteworthy from both an evolutionary perspective and in understanding the likelihood of an isolate to cause human disease. In terms of evolution, these two SNPs lend further support to the growing understanding of the evolutionary link between the chromosome and the plasmid. Both *toxB* and *tagA* are associated with the ability to cause human disease. The *toxB* gene promotes the production of type III secreted proteins which contributes to the adherence on epithelial cells (Tatsuno et al., 2001), while *tagA* [later renamed *StcE* (Lathem et al., 2002)] expression has been found to elicit a host immune response during human infection with STEC O157:H7 (Paton and Paton, 2002). This response is due to *tagA*/*StcE*’s ability to cleave C1 esterase inhibitor (C1-INH), which has been suggested as the cause of localized pro-inflammatory and coagulation responses resulting in tissue damage (Lathem et al., 2002). The SNP profile of *toxB* 7,453 G allele and the *tagA/stcE 1,438* G allele could be an indicator of a group of strains more likely to cause disease in humans, much like the *tir* 255 T found on the chromosome (Bono et al., 2007). The finding of the *tir*-linked SNPs in *toxB* and *tagA* further verifies that the pO157 plasmid plays an important role in the ability of a strain to cause disease in humans.

### Additional plasmids identified in *tir* 255 T allele and *tir* 255 A allele strains

The remaining non-pO157 plasmids identified were placed into one of 10 groups by nucleotide sequence and gene content similarity. Of the 10 groups, four plasmid groups were found in both *tir* 255 T allele and *tir* 255 A allele strains, while four plasmid groups were only found in *tir* 255 T allele strains and two plasmid groups in *tir* 255 A allele strains (Supplementary Table 9). The two 145 kb IncA/C plasmids in group 1 were identical in sequence and belonged to *tir* 255 T allele strains. These plasmids contained ARGs encoding chloramphenicol/florfenicol, tetracycline, trimethoprim, aminoglycoside, and sulfonamide resistance. They also contained the QacE delta 1 gene for quaternary ammonium compound resistance. These plasmids are also related to plasmids in *Klebsiella pneumoniae* (CP110604), *Shewanella algae* (CP032414), and *Salmonella enterica* subspecies *enterica* serovars Diarizonae (CP117189) and Typhimurium (CP100733).

The two 99 kb plasmids in group 2 differ by 4 SNPs and the location of an IS*3* family transposase. They belong to *tir* 255 A allele strains and the pO111 incompatibility type. These plasmids shared homology with bacteriophages that are chromosomally encoded (accession numbers NC_050153 and LM996300). Phaster identified the phage to be complete with a score greater than 90 with phage_Escher_RCS47_NC_042128 as the phage with the highest number of proteins most similar between the two.

The three plasmids in group 3 are 89 kb or 83 kb in size. The plasmids were found in *tir* 255 T allele strains and were the IncI1_I(Alpha) incompatibility type. The difference in size is due to deletions that include a colicin operon from the 83 kb plasmid. These plasmids contain a shufflon operon, an incomplete *tra* system, pilus relate genes, and colicin 1A. These plasmids are highly related to an unnamed plasmid from STEC O157:H7 strain ECP19-598 (CP0066754). They are also related to plasmids found in *Salmonella enterica* subspecies *enterica* serovars Agona (CP082483), Typhimurium (MW655523), and Derby (CP074320).

Group 4 plasmids belonging to the IncI2(Delta) incompatibility type are between 56 kb and 58 kb in size and were found one *tir* 255 A allele and three *tir* 255 T strains. The gene sequence order is conserved in this group of plasmids with the size differences related to deletions. The defining features of this plasmid group are pilus genes, incomplete *tra* operon, and a shufflon operon. The related plasmids from this group can also be found in other *E. coli* including strains 588888_1 (CP073614) and AR_0011 (CP024858).

Plasmids in group 5 ranging from 31 kb to 43 kb in size were identified in nine strains with four being *tir* 255 A allele strains and five *tir* 255 T allele strains. Two strains, EC4115 and pMB9_3, belonged to the pEC4115 incompatibility type, while the remaining seven were unclassified. The size difference between these plasmids can be associated with IS elements and deletions. The plasmids of group 4 contain some of the *tra* genes that are associated with transferring plasmids from one strain to another. Plasmids belonging to this group are not only found in different *E. coli* but are also found in *Salmonella enterica* strains.

Group 6 plasmids range in size from 6,675 bp to 7,324 kb and occurred in three *tir* 255 T alleles and one *tir* 255 A allele strain. The incompatibility type of these plasmids is unclassified. There are 51 SNP differences between this group of plasmids with most found in a hypothetical gene and its upstream and downstream untranslated region. This group of plasmids is similar to the pColD157 plasmid isolated from EHEC O157:H7 strain CL40cu (Y10412) with the defining feature being the colicin D gene with its immunity and lysis genes.

Group 7 was comprised of a single plasmid sequence of 6,402 bp found in three *tir* 255 A allele strains and an unclassified incompatibility type. These plasmids contained five hypothetical proteins, type III toxin-antitoxin system, and several genes associated with plasmid mobilization. The plasmids share the same gene arrangement as plasmid pEH09-18-41_4 from *E. coli* EH09-18-41 (CP063507) but differ by 37 SNPs located mainly in a nuclease gene.

The 3,306 bp plasmids in group 8 were first identified in the Sakai strain and named pOSAK1. pOSAK1 has an unclassified incompatibility type and was only found in *tir* 255 T allele strains. The plasmids are identical except for one plasmid having a SNP difference from the other plasmids.

The three plasmids in group 9 vary in size between 2,679 bp and 4,634 bp in size and have an unclassified incompatibility. Two were found in *tir* 255 A allele strains and one in a *tir* 255 T allele strain. Group 10 contains two 2.7 kb plasmids found in *tir* 255 A allele strains. These plasmids contain three genes annotated as encoding two hypothetical genes and a plasmid recombination enzyme gene. There were no plasmids in the GenBank non-redundant nucleotide database that had a similar sequence to these plasmids.

There were two miscellaneous plasmids that were not placed in any of the 10 groups. Both plasmids, 1,538 bp and 93,970 bp in size, were found in STEC strain NE1092-2. The 1,538 bp plasmid belongs to incompatibility type IncC and is closely related to plasmids found in *E. coli* strain Iso00041 (CP095159) and *Shigella flexneri* 1b strain A-10383 (CP130069). The 93 kb plasmid was found in a *tir* 255 T allele strain with the incompatibility type Col(MG828). This plasmid contains two aminoglycoside resistance genes along with genes encoding for chloramphenicol/florfenicol, tetracycline, and sulfonamide resistance. This plasmid also contains a mercury resistance operon. This plasmid has regions of homology to plasmids in *Klebsiella pneumoniae* (CP129795, CP124165, and CP124154) and *Phytobacter diazotrophicus* (AP028052).

## Conclusion

The goal of this study was to identify genomic differences between *tir* 255 T allele and *tir* 255 A allele strains and determinants that could be responsible for the different *tir* 255 allele associations with human disease. The most accurate way to achieve that was with complete or nearly complete closed genomes. We used 57 completely closed genomes and another that was complete except for the chromosome being in two contigs for this study. STEC O157:H7 genomes are constantly being sequenced and added to the public databases; therefore, new unique genes or mobile elements may be discovered in future as part of the STEC O157:H7 pangenome. However, the differences identified between *tir* 255 T allele and *tir* 255 A allele core genomes in this study were established with strains that were from five different countries and that were well separated in time and space. Consequently, the core *tir* 255 T and A allele genomes will likely not fluctuate as much as the pangenome in future with the addition of new genomes.

The chromosome and pO157 plasmid from *tir* 255 T allele and *tir* 255 A allele strains differed in size and the average number of CDS from each other. The size differences in the chromosome were not due to additional mobile elements such as prophage or IS elements. However, there were differences between the types of annotated prophage between *tir* 255 T and A allele strains, including allele-specific prophages that were grouped by strain relatedness. This suggests that prophages have remained integrated into the chromosome long enough to become part of a clonal lineage. The pO157 plasmid size differences could be attributed to IS elements with the *tir* 255 A allele strains having on average four more IS elements than the *tir* 255 T allele strains. There were also compelling SNPs on pO157 that were in linkage disequilibrium with the *tir* 255 alleles.

The overall percentage of STEC O157 strains that carried ARGs was quite low, with resistance genes observed on the chromosome in *tir* 255 A allele strains but three ARGs carrying plasmids in *tir* 255 T allele strains. Certain plasmid sequences were unique to either the *tir* 255 A or T allele strains. However, the allele-specific plasmids were not found in all allele strains suggesting additional plasmids are not likely responsible for the association of *tir* 255 T variants having a greater probability of causing disease in humans.

Within the core chromosomes of *tir* 255 A and T strains, 18 CDS were unique to *tir* 255A and 3 were unique to *tir* 255 T. SNP alleles consistently observed in the *tir* 255A lineage resulted in amber mutations in four genes (*glpQ*, *eutA*, *apqZ*, and *lpfE*). Out-of-frame indel alleles linked to the *tir* 255A lineage were also identified in *scpB* and *scpC* genes. While the inactivated genes were predicted to provide a growth advantage, none necessarily explain why *tir* 255 A allele strains are less likely to cause disease in humans than *tir* 255 T allele strains without additional research. The results presented provide a more complete understanding of the genomics of this important public health pathogen and new insight into genomic features that may contribute to decreased association with human disease. Further investigation using transcriptomic and metabolomics methods, phenotypic antimicrobial resistance profiles, and disease models to assess the role of the variants may provide a greater explanation for the *tir* 255 allele differences in association with human disease.

## Data availability statement

The datasets presented in this study can be found in online repositories. The names of the repository/repositories and accession number(s) can be found in the article/Supplementary material.

## Author contributions

MW: Conceptualization, Data curation, Formal Analysis, Writing – original draft. MC: Formal Analysis, Writing – original draft. GH: Formal Analysis, Writing – review & editing. ME: Formal Analysis, Writing – review & editing. DH: Formal Analysis, Writing – review & editing. TS: Formal Analysis, Writing – review & editing. JB: Conceptualization, Data curation, Formal Analysis, Project administration, Supervision, Writing – original draft.
